# In this month’s Bulletin

**DOI:** 10.2471/BLT.21.000121

**Published:** 2021-01-01

In the editorial section, James Campbell and Fahrettin Koca (2) discuss the support needed for the health and care workforce highlighted by the COVID-19 pandemic. Jozef Bartovic et al. (3) point to the need for equitable access to vaccines for refugees and migrants.

In the news section, Lynn Eaton (6–7) reports on how pandemic-response measures, including school closures and movement restrictions, are impacting the sexual and reproductive health and rights of adolescent girls. Fadi Meroueh talks to Andréia Azevedo Soares (8–9) about ensuring health equity and harm reduction services in prisons.

## Afghanistan, Armenia, Bangladesh, Bhutan, Eritrea, Ethiopia, Ghana, India, Islamic Republic of Iran, Iraq, Kenya, Lesotho, Malawi, Maldives, Namibia, Nepal, Niger, Pakistan, Rwanda, Sri Lanka, Sudan, Syrian Arab Republic, Uganda, United Republic of Tanzania and Zimbabwe.

### Improving neonatal outcomes

Tedbabe Degefie Hailegebriel et al. (69–71) follow implementation of kangaroo mother care.

## China

### Eliminating mother-to-child transmission of hepatitis B virus

Zheng Hui et al. (10–18) model infection control measures.

## Malawi

### Ensuring accurate translation

Aisha Nandini Zoe Dasgupta (67–68) analyse terms used to refer to contraception.

## Republic of Korea 

### National triage of COVID-19 patents

Kyung-Bok Son et al. (62–66) report the severity classification used to group patients.

## South Sudan

### Preventing tuberculosis

Andrew T Boyd et al. (34–40) study measures to prevent HIV/TB coinfection.

## Viet Nam 

### Surviving out-of-hospital cardiac arrest

Son N Do et al. (50–61) study outcomes in a prospective cohort.

## Global

### Analysing available seroprevalence data

John PA Ioannidis (19–33) estimates the infection fatality rate of COVID-19.

### Reducing sugar production and consumption

Anne Marie Thow et al. (41–49) discuss implications for farmers.

